# Distinct Modulated Pupil Function System for Real-Time Imaging of Living Cells

**DOI:** 10.1371/journal.pone.0044028

**Published:** 2012-09-04

**Authors:** Tomonobu M. Watanabe, Yoshikazu Tsukasaki, Hideaki Fujita, Taro Ichimura, Tatsuya Saitoh, Shizuo Akira, Toshio Yanagida

**Affiliations:** 1 RIKEN Quantitative Biology Center (QBiC), 6-2-3 Furuedai, Suita, Osaka, Japan; 2 Graduate School of Frontier Biosciences, Osaka University, 1–3 Yamadaoka, Suita, Osaka, Japan; 3 World Premier International Research Center Initiative, Immunology Frontier Research Center, Osaka University, 1–3 Yamadaoka, Suita, Osaka, Japan; 4 Department of Host Defense, Research Institute for Microbial Diseases, Osaka University, 1–3 Yamadaoka, Suita, Osaka, Japan; 5 PRESTO, Japan Science and Technology Agency, 4-1-8 Honcho, Kawaguchi, Saitama, Japan; Tufts University, United States of America

## Abstract

Optical microscopy is one of the most contributive tools for cell biology in the past decades. Many microscopic techniques with various functions have been developed to date, i.e., phase contrast microscopy, differential interference contrast (DIC) microscopy, confocal microscopy, two photon microscopy, superresolution microscopy, etc. However, person who is in charge of an experiment has to select one of the several microscopic techniques to achieve an experimental goal, which makes the biological assay time-consuming and expensive. To solve this problem, we have developed a microscopic system with various functions in one instrument based on the optical Fourier transformation with a lens system for detection while focusing on applicability and user-friendliness for biology. The present instrument can arbitrarily modulate the pupil function with a micro mirror array on the Fourier plane of the optical pathway for detection. We named the present instrument DiMPS (Distinct optical Modulated Pupil function System). The DiMPS is compatible with conventional fluorescent probes and illumination equipment, and gives us a Fourier-filtered image, a pseudo-relief image, and a deep focus depth. Furthermore, DiMPS achieved a resolution enhancement (pseudo-superresolution) of 110 nm through the subtraction of two images whose pupil functions are independently modulated. In maximum, the spatial and temporal resolution was improved to 120 nm and 2 ms, respectively. Since the DiMPS is based on relay optics, it can be easily combined with another microscopic instrument such as confocal microscope, and provides a method for multi-color pseudo-superresolution. Thus, the DiMPS shows great promise as a flexible optical microscopy technique in biological research fields.

## Introduction

Imaging of living biological specimens has greatly advanced through the development of several microscopic techniques. For example, a phase contrast microscope, which converts small phase shifts of the light passing through a transparent specimen into contrast changes, has been widely used for the observation of cell morphology [1]. A differential interference contrast (DIC) microscope is used to enhance the periphery of specimen through the interferometry principle to gain information regarding the optical path length [2]. The DIC microscope can obtain a high-contrast image without a bright diffraction halo seen in the phase contrast microscope. Fluorescence microscopy has been a powerful tool in the past decades along with the development of fluorescent probes such as organic dye and green fluorescent protein (GFP) [3]–[6]. Positional information (localization and movement) of a target protein fused with a fluorescent probe can be obtained for living cells and/or tissues by using the fluorescent microscope [7]. There are various fluorescent microscopic techniques for observing various kinds of biological events, i.e., confocal microscopy for obtaining optical sections of thick samples [8], two photon microscopy for deeper imaging [9], [10], superresolution microscopy for observing nano-structures [11], etc. A person who is in charge of a project has to choose a microscope with these uses considering which one is the most appropriate for their experiment, and in most cases, the use of multiple techniques is required. This makes biological assays time-consuming and expensive. To solve this problem, we here developed a microscopic system involving distinct microscopies in one instrument focusing on its usability and applicability for biological observations.

To modulate an image under an optical microscope, there is a technique based on the optical Fourier transformation with a lens system for detection [12]–[14]. Because the point spread function (PSF) of an optical system is the power spectrum of the pupil function on the Fourier plane, modulation of the pupil function causes modulation of the image. For example, when the outer rim of the pupil function is masked, the PSF is blurred (apodizing-masked) [14]. On the other hand, on masking of the center of the pupil function, the PSF shrinks, which results in the improvement of the spatial resolution (superresolving-masked) [13]. Based on these effects of modulation of the pupil function, we have developed a novel optical microscopic system, named DiMPS (Distinct optical Modulated Pupil function System), and tested it on biological samples. The DiMPS involves micro mirror arrays placed on the Fourier plane in the detection pathway, but without scanning equipment. In this paper, we describe the basic DiMPS design and its construction, and demonstrate its potential for live cell imaging.

## Results

### Construction of DiMPS

**Figure 1:**
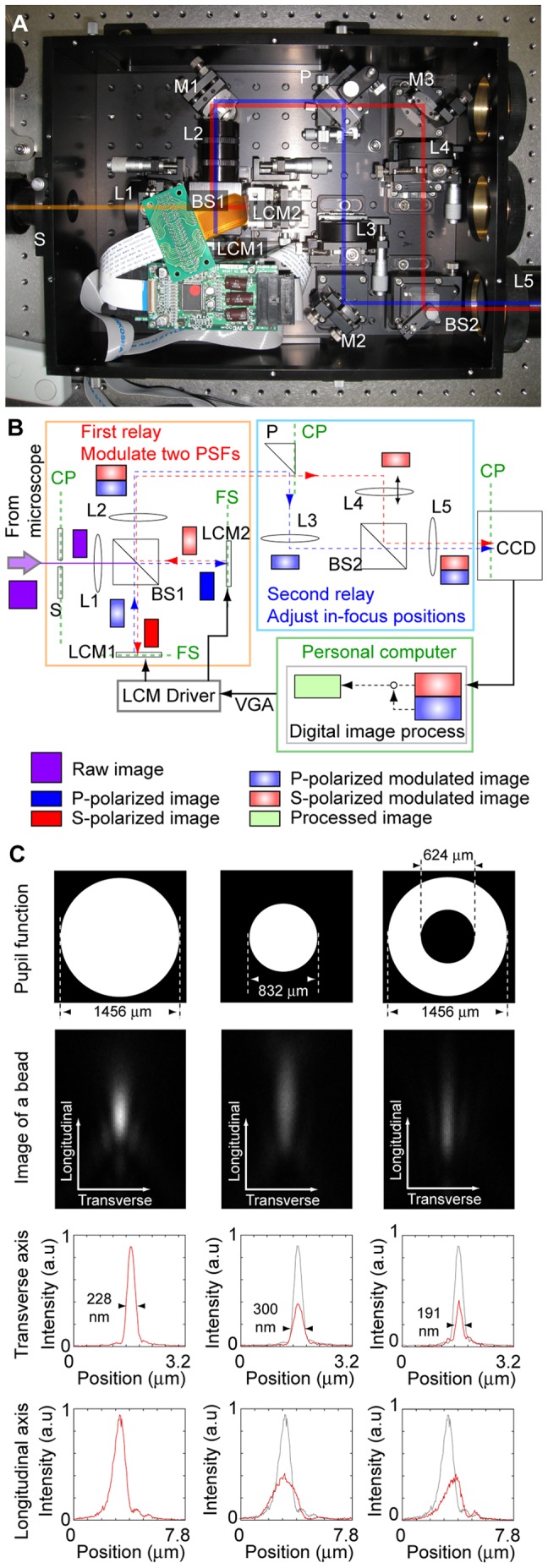
Basic of the DiMPS. (A) Photograph of the DiMPS. (B) Schematic drawing of the DiMPS. S, spatial mask; BS, polarizing beam splitter; LCM, liquid crystal micro mirror array; P, prism mirror; L, lens; FP, Fourier plane; CP, conjugate plane. See methods for the optics construction. (C) Typical modulations of the pupil function. The pupil function is the polarization pattern in the LCM. Black part shows where the light is blocked. The PSF is represented by the fluorescence distribution of a φ100 nm bead with an emission peak at 515 nm (Invitrogen). Red lines show the one-dimensional fluorescence intensity profile of the PSF along the transverse and longitudinal axes. Gray lines are the one-dimensional fluorescence intensity profile of a normal PSF. Scale bars, 1 µm.

We constructed an optical system to obtain two images having distinct pupil functions that were arbitrarily modulated (Fig. 1*A, B*, and see materials and methods). The DiMPS has two optic relays. The first relay is for splitting the image into two polarized images and for modulating the pupil function of each. An image passes through a spatial mask positioned at the imaging plane to reduce the field of view by half (S in Fig. 1 *A* and *B*). The optical pathway is split into two by a polarizing beam splitter before the Fourier plane (BS1). Reflective LCOS (Liquid Crystal On Silicon) micro mirror arrays (LCMs) are set on two of the Fourier planes of lens L1. Same mask pattern was used for both of LCMs to generate two images with opposite polarization, except for siDiMPS where two images were subtracted to generate one resolution enhanced image (see below). The polarization state of each pixel can be controlled by using a video graphics array signal from a computer at a refresh rate of 60 Hz. The light whose polarization has been modulated by the LCMs is transmitted through or reflected by the polarizing beam splitter. The system composed of the LCMs and the beam splitter can modulate only the amplitude but not the phase. A phase spatial light modulator based on LCOS technology is also available for similar equipment, however, it is not suitable for fluorescent multi-color imaging because of its strong wavelength dependency. The applicability for multi-color imaging is necessary for the recent biology. Because we here had focused on applicability and user-friendliness in vast cases of biological assay, the amplitude modulation, which has the low wavelength dependency such as one for a video projector, is preferred. Its lower price is another reason that amplitude modulation is preferable for practical use. The second relay is for optimizing the in-focus positions of the two images [15]. Moving either L3 or L4 along the optical axis changes the optical length between the two lenses, resulting in a difference in the in-focus positions. Finally, the images at two pathways are combined into one image, or projected side by side onto a charge-coupled device (CCD) camera by a second polarizing beam splitter (BS2). When the specimen had an anisotropic polarization property such as a birefringent sample, a quartz depolarizer was set at the back of the objective to negate any effect of the polarization.

To examine the effect of pupil function modulation using the DiMPS, we observed a fluorescence image of a fluorescent bead of 100 nm in diameter as a PSF (Fig. 1*C* and Fig. S1). The longitudinal (Z) section was obtained by sequential scanning at 50 nm interval. The typical shape of the pupil function is a full circle. In our case, the diameter of the pupil on the surface on an LCM is approximately 1.5 mm with a 150× objective. Decreasing the diameter of the pupil function blurred the fluorescence image of the bead in both the longitudinal (optical) and transverse (horizontal) axes (Fig. 1*C*, *center*). Meanwhile, masking of the center of the pupil function shrunk the image (Fig. 1*C*, *right*) down to 180 nm at minimum (Fig. S1, *top-left*). We can apply arbitrary pupil patterns on the LCMs and the fluorescence images are modulated in correspondence to these pupil functions (Fig. S1, *lower*). Thus, though masking of the pupil function decreases the signal intensity from a specimen, the image can be easily modulated.

### Fourier High-pass Filtering and Extension of Focal Depth with the DiMPS

**Figure 2:**
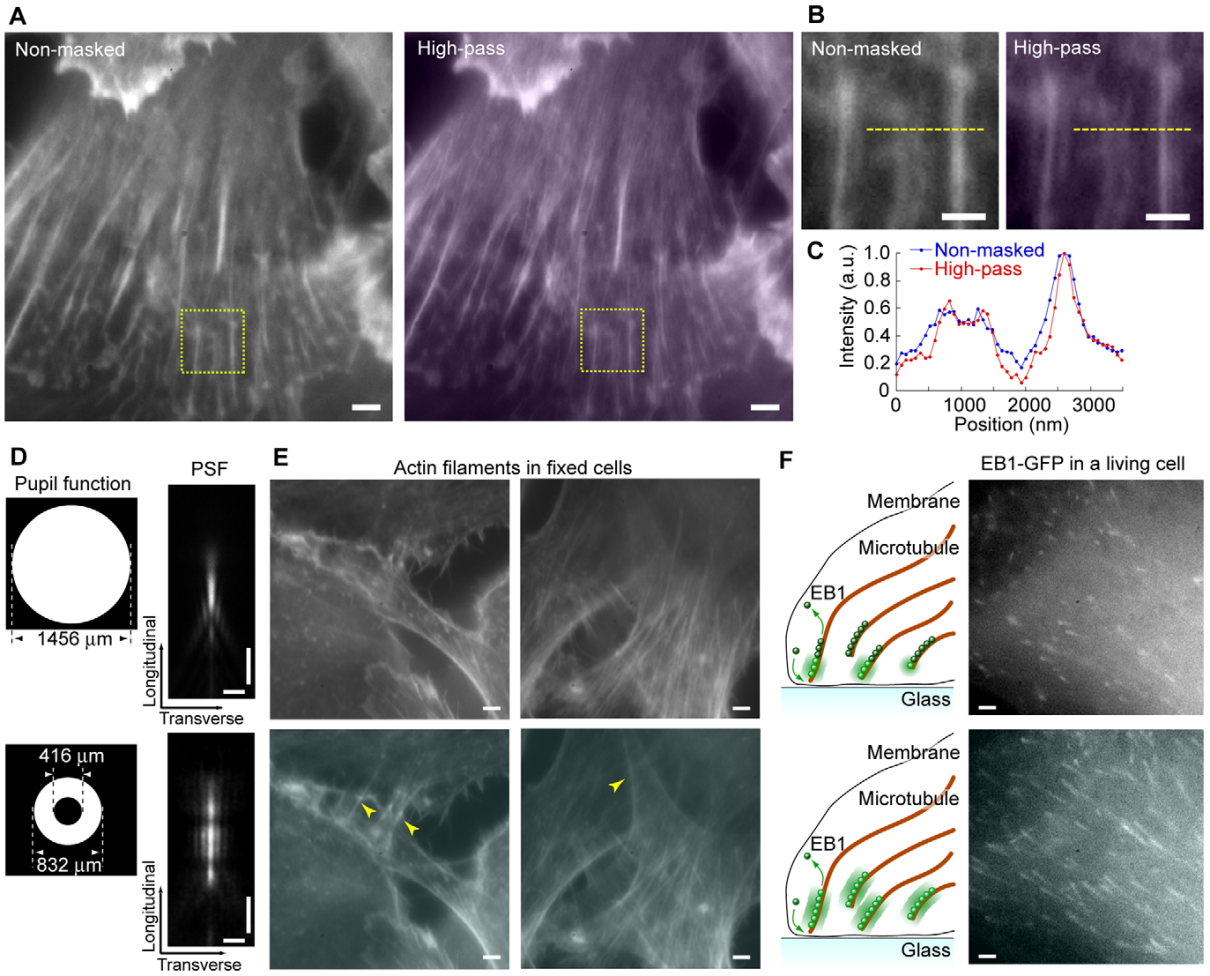
Fluorescent imaging with the DiMPS. (A) Fluorescent images of actin bundles labeled with Alexa488 phalloidin in a HeLa cell. Left, non-masked image. Right, high-pass filtered image with a superresolving-mask. Scale bars, 2 µm. (B) Enlarged images of the yellow rectangles in **A**. Scale bars, 1 µm. (C) One-dimensional fluorescence intensity profile of the yellow lines in **B**. Blue, non-masked. Red, superresolving-masked. (D) Extended PSF. Left panels, the pupil functions used. Right panels, the PSFs. The PSF is the fluorescence distribution of a φ100 nm fluorescent bead (right panels). Scale bars, 1 µm. (E) Two typical fluorescent images of actin bundles in overlapping cells. Upper and lower panels are non-masked images and ones obtained with the extended PSF, respectively. Yellow arrowheads indicate the points that are not visible with the normal PSF but are with the extended one. Scale bars, 2 µm. (F) Observation of GFP-fused EB1 in a living KPL4 cell. Upper and lower panels are non-masked images and ones obtained with the extended PSF, respectively. Left panels, schematic drawing showing how EB1 is illuminated. Right panels, snapshots of the EB1 observation. The movies are available as supplementary material (movies S1 and S2). Scale bars, 2 µm.

The spatial resolution of an optical microscope is significantly constrained by optical diffraction limits [16]. To overcome this limitation, a number of techniques have been developed that are called superresolution [11]. The DiMPS achieves superresolution-like effect by masking the center of the pupil function (superresolving-masked) [13], though the effect is smaller than recent superresolution techniques. The spatial resolution of a microscope is usually defined as the full width at half maximum (FWHM) of the PSF profile. When using a ring with an inner diameter of 624 µm and an outer diameter of 1456 µm as a pupil function, the FWHM is improved to 190 nm from 230 nm by the high-pass filtering effect at the Fourier plane (Fig. 1*C*, *right*). Note that, there is a trade-off between the improvement of FWHM and the intensity, which is directly related to the signal to noise (S/N) ratio of the image, for example, the intensity is decreased 10-fold to obtain 180 nm spatial resolution (Fig. S2). The LCM used here, whose contrast ratio is 1000∶1, cannot absolutely block 100% of the light. The reflection efficiency of the beam splitter used was not also 100% (>90% from 500 nm to 650 nm wavelength) while the transmission was almost 100%. Therefore, when the area of the mask become too large, the light that was not blocked become dominant elements than one that was transmitted and the improvement of FWHM is diminished (Fig. S2, *right*). To confirm the effect of the superresolving-mask on cell imaging, we observed actin bundles labeled with Alexa488-phalloidin in a fixed cell which had enough intensity to compensate for the S/N ratio decrease (Fig. 2*A and B*). The actin bundles were more clearly observed by the high-pass filtering effect with the superresolving-mask. The fluorescence intensity profile of the images shows the obvious improvement of the spatial resolution with the superresolving-mask (Fig. 2*C*). Thus, we can easily obtain a resolution enhanced image by using DiMPS without the use of other equipment.

The DiMPS also enables one to achieve an extended PSF on the longitudinal axis, which increases the focal depth [17] (Fig. 2*D*). The focal depth of the extended PSF in our case was roughly 3 µm, while that under the normal condition is about 1 µm. When observing the place where cells overlapped, the DiMPS enable us to observe both cells using the extended PSF (Fig. 2*E*). This ability of DiMPS to give an extended PSF is applicable to live cell imaging. To confirm the effect of the extended PSF on live cell imaging, we performed live cell imaging of GFP-labeled EB1, which is one of the microtubule-binding proteins targeting the plus ends of polymerizing microtubules [18]–[20] (Fig. 2*F*, and movies S1 and S2). While the fluorescent image of EB1 usually forms a comet shape [19], [20], the length of the comet is estimated to be slightly shorter because of the thin focal depth on conventional fluorescent microscopy (Fig. 2*F*, *upper*). In contrast, the DiMPS with an extended PSF visualized more EB1-comets, and each comet was longer than that observed with a conventional microscope (Fig. 2*F*, *lower*). Thus, by using the DiMPS, microscope user can obtain various effects on the image in real time and on site just by changing the pupil function.

### Subtractive Imaging with the DiMPS (siDiMPS) for Resolution Enhancement

**Figure 3:**
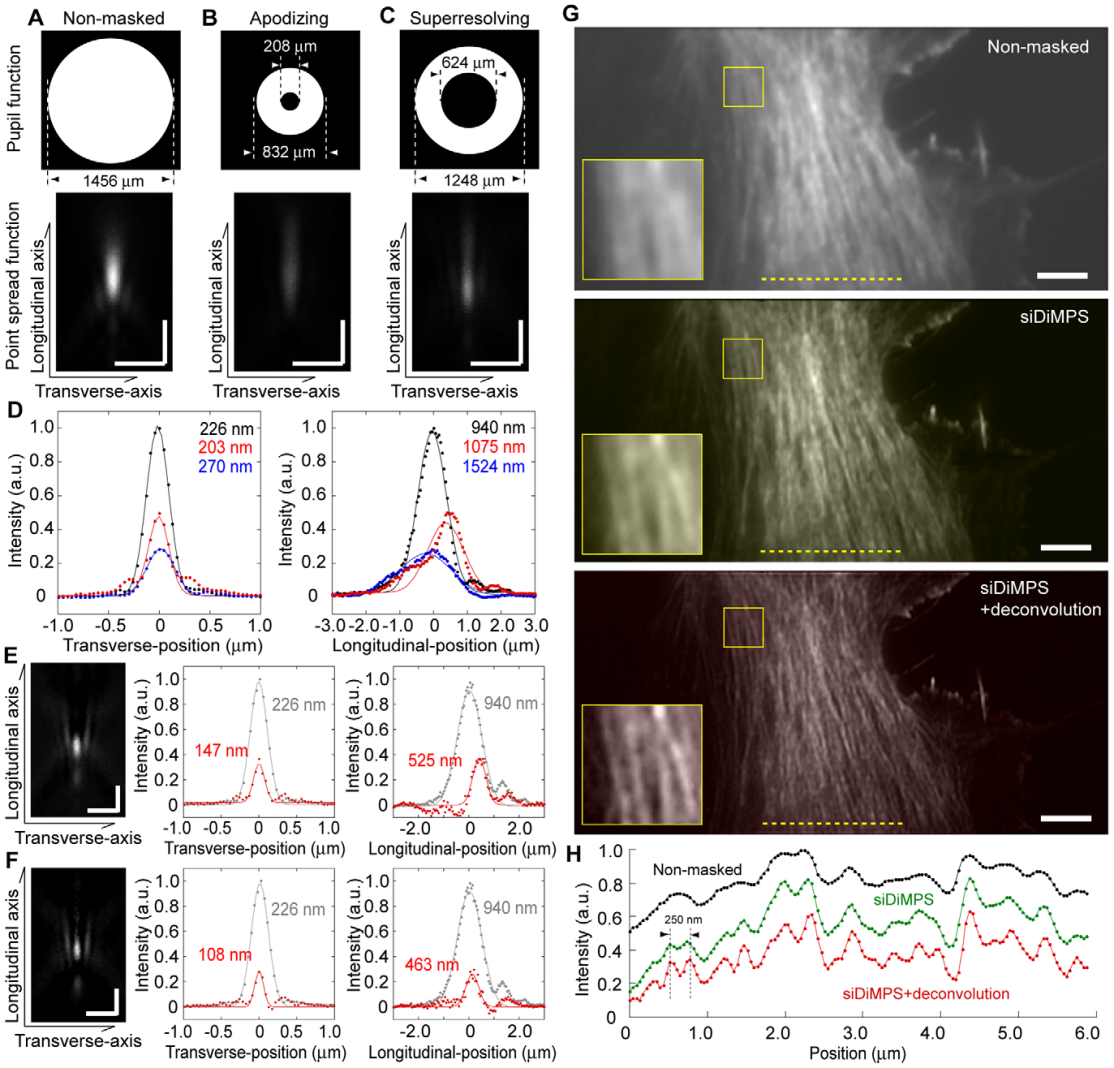
Resolution enhancement with the siDiMPS. (A, B, C) Pupil functions and fluorescence distributions of a φ100 nm bead with a non-masked (A), apodizing-masked (B), and superresolving-masked PSF (C). Upper panels, pupil functions; lower panels, PSF images in the longitudinal-transverse plane. (D) One-dimensional fluorescence intensity profiles of the non-masked (black), apodizing-masked (blue), and superresolving-masked PSF (red). Values indicate spatial resolution determined at FWHM. (E, F) Fluorescence distributions determined by subtracting an apodizing-masked PSF from a superresolving-masked one without (E), and with optimizing symmetry of PSF by adjusting lens L4 position along the light path (F). Left panels, PSF images in the longitudinal-transverse plane; middle and right panels, one-dimensional fluorescence intensity profile of each PSF along the transverse axis (middle) and longitudinal axis (right), respectively. Values indicate spatial resolution determined at FWHM. Red and gray lines are one dimensional fluorescence profiles of the non-masked (E) and extended PSF (F), respectively. All scale bars, 1 µm. (G) siDiMPS image of Alexa488-phalloidin stained actin bundles in a fixed cell. Top, non-masked. Middle, siDiMPS. Bottom, siDiMPS+deconvolution. Inserts, magnifications of the areas in yellow rectangles, respectively. Scale bars, 2 µm. The images were obtained with a 100 ms exposure time. (H) One-dimensional fluorescence intensity profiles of the yellow broken lines in G (black, conventional; green, siDiMPS; red, siDiMPS+deconvolution).

We have shown the effect of modulation of the pupil function so far. Here, we demonstrate an application involving image computation with two images acquired by distinctly modulating the pupil functions; i.e., a resolution enhancement method with which the spatial resolution can be further enhanced by subtracting the apodizing-masked image from the superresolving-masked image, similar to the subtraction performed for a confocal pair with different pinhole sizes [21]. The only requirement for better resolution is to identify two images with distinct PSF profiles such that the subtracted PSF is as narrow as possible. Because of various aberrations in the optics including the objective, it is difficult to precisely pre-calculate the PSF shape. Moreover, the effect of contrast ratio of the LCMs, which, is appeared when the reflective area is small (Fig. S2), also complicates the pre-calculation. Therefore, we sought the optimal pupil function by actual measurements since the DiMPS can easily produce a huge number of the mask pattern. By testing more than 200 pairs of images, we identified the best combination of pupil functions to achieve the narrowest PSF that is defined by the fluorescence distribution of a fluorescent bead of 100 nm in diameter (Fig. 3, *A–F*). Although outer rim of the Fourier plane contains high spatial information of the image, we noticed that PSF elongates in the longitudinal direction when pupil function of large diameter ring was used (Fig. S1, *top left*). This is probably due to the large effect of the aberration in this area, and worsens the resolution along the longitudinal axis. Thus, the best mask we used consists of the mask that blocks both inner and outer area of the Fourier plane. The best resolution was achieved with two ring-shaped masks; one with an inner diameter of 208 µm and an outer diameter of 832 µm (Fig. 3, *B* and *D*, *blue*), and the other with an inner diameter of 624 µm and outer diameter of 1248 µm (Fig. 3 *C* and *D*, *red*), the FWHM of the subtracted PSF improved from 230 nm to 150 nm along the transverse axis, and from 940 nm to 530 nm along the longitudinal (optical) axis (Fig. 3 *E*). The in-focus positions of the two pathways were unmatched, most likely due to spherical aberrations (Fig. 3 *D*, *right panel*). By moving lens L4 in Fig. 1, we can adjust the in-focus positions of the two images, which are defined as where the contrast is highest, independently [15]. This adjustment was made so that the subtracted PSF was narrower (Fig. 3 *F*). In the present study, we achieved a spatial resolution of 110 nm along the transverse axis and 460 nm along the longitudinal one, respectively. We named this method siDiMPS (subtractive imaging of DiMPS). An obtained image consists of a superresolving-masked image (upper) and an apodizing-masked one (lower) in 512×512 pixels (22×22 µm^2^) (Fig. S3 *A*). The non-weighted subtracted image of the two images was displayed on site in real time (Fig. S3 *B*). When observing an image of actin bundles labeled with Alexa488-phalloidin in fixed mouse fibroblasts cells, the siDiMPS not only enabled us to obtain a resolution-enhanced image but also decreased the background fluorescence since the resolution along both the transverse and longitudinal axes was improve (Fig. 3 *G* and *H*). Additionally applying the deconvolution process [22], we could obtain the clearer image (Fig. 3 *G, bottom*), and it was possible to individually discriminate two actin bundles within 250 nm that is close to the resolution limit of the conventional microscope (Fig. 3 *H*, *red*).

### GFP Live Observation Using siDiMPS

**Figure 4:**
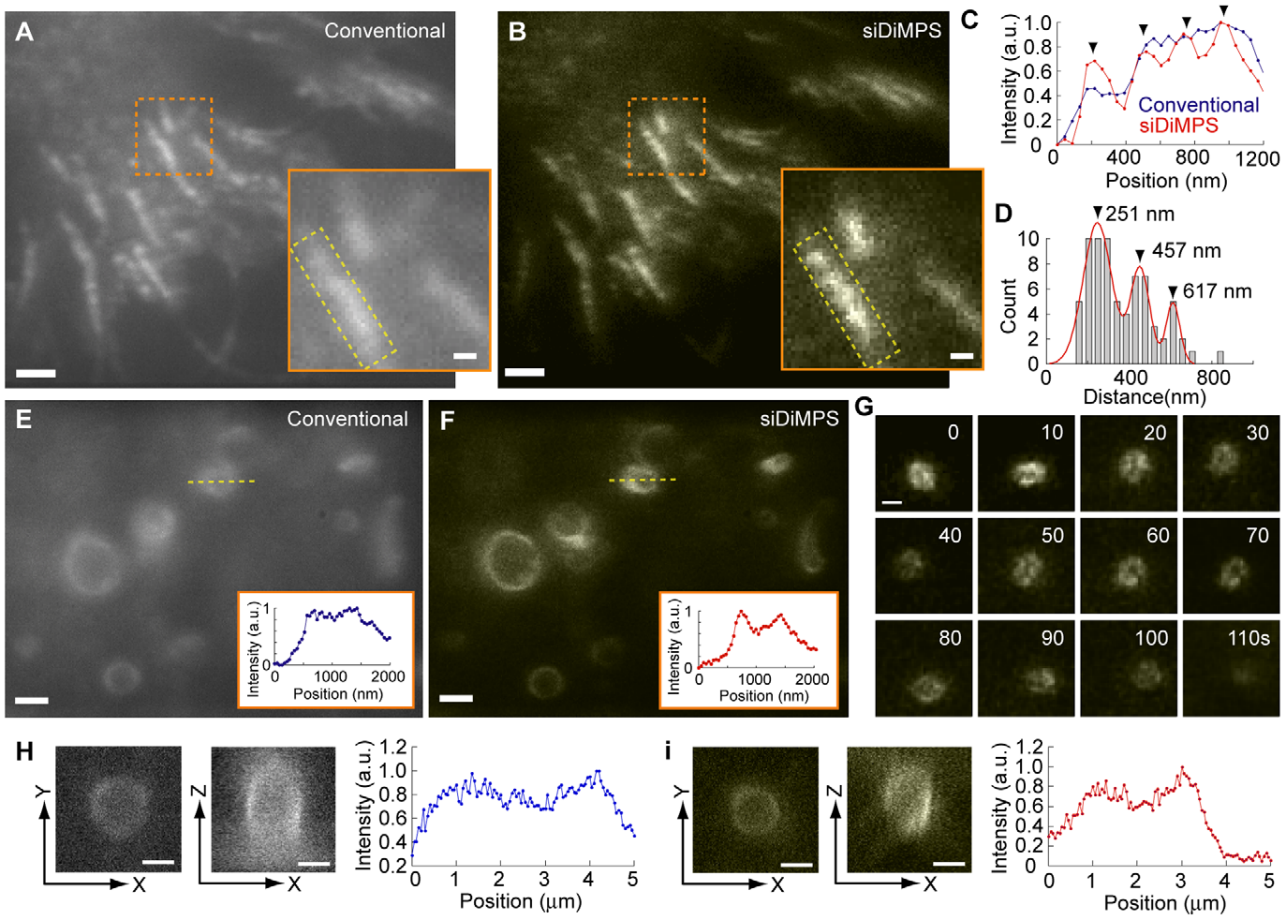
Biological applications of siDiMPS using green fluorescent protein. (A,B) Fluorescence images of localized E-cadherin-GFP in a living cell obtained by conventional microscopy (A) and siDiMPS (B). Scale bars, 1 µm. The exposure time for the camera was 30.28 ms. The images shown are the averages of 100 acquired images (total frame rate, 0.3 Hz). Inserts, magnifications of the areas in orange rectangles, respectively. Scale bars, 200 nm. (C) One-dimensional fluorescence intensity profiles inside the yellow rectangle in A (blue, conventional) and B (red, siDiMPS). Arrowheads indicate the periodic peaks of fluorescence intensity. (D) Histogram of the distance between two peaks within the periodic localization of E-cadherin-GFP. Arrowheads and values represent the peaks in the histogram. (E, F) Fluorescence images of autophagosomes labeled with GFP-LC3 in a living cell acquired by conventional microscopy (E) and siDiMPS (F). Scale bars, 1 µm. Inserts, the intensity profiles of the one-dimensional fluorescence intensity profile (yellow line) in each panel. (G) siDiMPS time lapse images of a small organelle labeled with GFP-LC3. Scale bar, 400 nm. The exposure time for the camera was 30.28 ms. Images E, F and G are averages of 10 acquired images (total frame rate, 3 Hz). (H, I) Three dimensional images of autophagosome acquired by conventional microscopy (H) and siDiMPS (I). Left and middle panels are XY-image and XZ-image, respectively. Right panels are one-dimensional fluorescence intensity profile along longitudinal axis (Z) at the center of the images. Scale bars, 1 µm. The exposure time for the camera was 100 ms.

To further demonstrate the effect of siDiMPS, we observed the localization of E-cadherin fused with GFP in a living cell. E-Cadherin is a calcium-dependent cell adhesion molecule that plays important roles in cell morphology and architecture in a wide variety of cells, and its loss promotes tumor progression [23], [24]. A cell that overexpresses E-cadherin-GFP was seeded onto a glass surface coated with purified E-cadherin to mimic cell-cell adhesion [25]. SiDiMPS enabled us to observe the fiber-like structure of E-cadherin more clearly than under a conventional microscope (Fig. 4, *A* and *B,* and movie S3). Furthermore, the background fluorescence, which was most likely caused by E-cadherin-GFP in the cytosol, was dramatically reduced due to the subtraction of low-spatial frequency image. The fiber-like structure resembled a string of beads composed of small E-cadherin. This bead-like localization was more clearly observed in the siDiMPS image (Fig. 4, *A* and *B*, *insertions,* and *C*). A histogram of the distances between localizations showed peaks at 251, 457 and 617 nm, suggesting that cadherin clusters were arranged every ∼200 nm (Fig. 4 *D*), which is in good agreement with that observed with other nanoscopic methods [26], [27]. This distance (∼200 nm) is similar to the sizes of the compartmentalization structures or actin meshwork along the cytoplasmic surface of the plasma membrane [28]–[30]. The siDiMPS enabled us to obtain the resolution-enhanced image where E-cadherin clusters are arranged along actin fibers underneath the membrane in a living cell.

E-Cadherin observations were limited to the juxtamembrane region underneath the glass surface. To further demonstrate the potential of siDiMPS for intracellular imaging, we observed autophagosomes containing GFP-tagged microtubule associated protein light chain 3 (LC3) [31]–[33]. Autophagy is a catabolic process comprising degradation of cytoplasmic constituents via the lysosomal machinery and is induced by starvation [31], [33]. At 30 minutes after starvation in a mouse embryonic fibroblast (MEF) cell, many organelles labeled with GFP-LC3 were observed intracellularly under a fluorescence microscope (Fig. 4 *E*). SiDiMPS emphasized the periphery of organelles that were most likely autophagosomes (Fig. 4 *F* and movie S4). Time-lapse imaging showed that a small, 300 nm diameter, organelle was moving and changing in shape in a manner similar to an amoeba (Fig. 4 *G* and movie S5). Though most GFP-LC3 labeled organelles over 1000 nm had a round shape, which is also indicative of autophagosomes, amoeba-like organelles were occasionally observed among them (movie S6). To confirm the resolution enhancement along the longitudinal axis, we compared the three dimensional image of the autophagosome obtained by Z-scan at 50 nm interval, and compared images taken by conventional microscope and siDiMPS (Fig. 4, *H* and *I*). The sectioning effect along the longitudinal axis was clearly confirmed by the siDiMPS image. Furthermore, siDiMPS can obtain movies with the resolution enhanced. The images taken at 300 ms gave us to observe unidirectional movement of the small autophagosomes (movies S7). Thus, siDiMPS can be used to simultaneously observe structural changes and movements of small organelles in living cells in real time, which would be advantageous for interpreting the observed phenomenon.

### Resolution Enhancement at High Frame Rate Using siDiMPS

**Figure 5:**
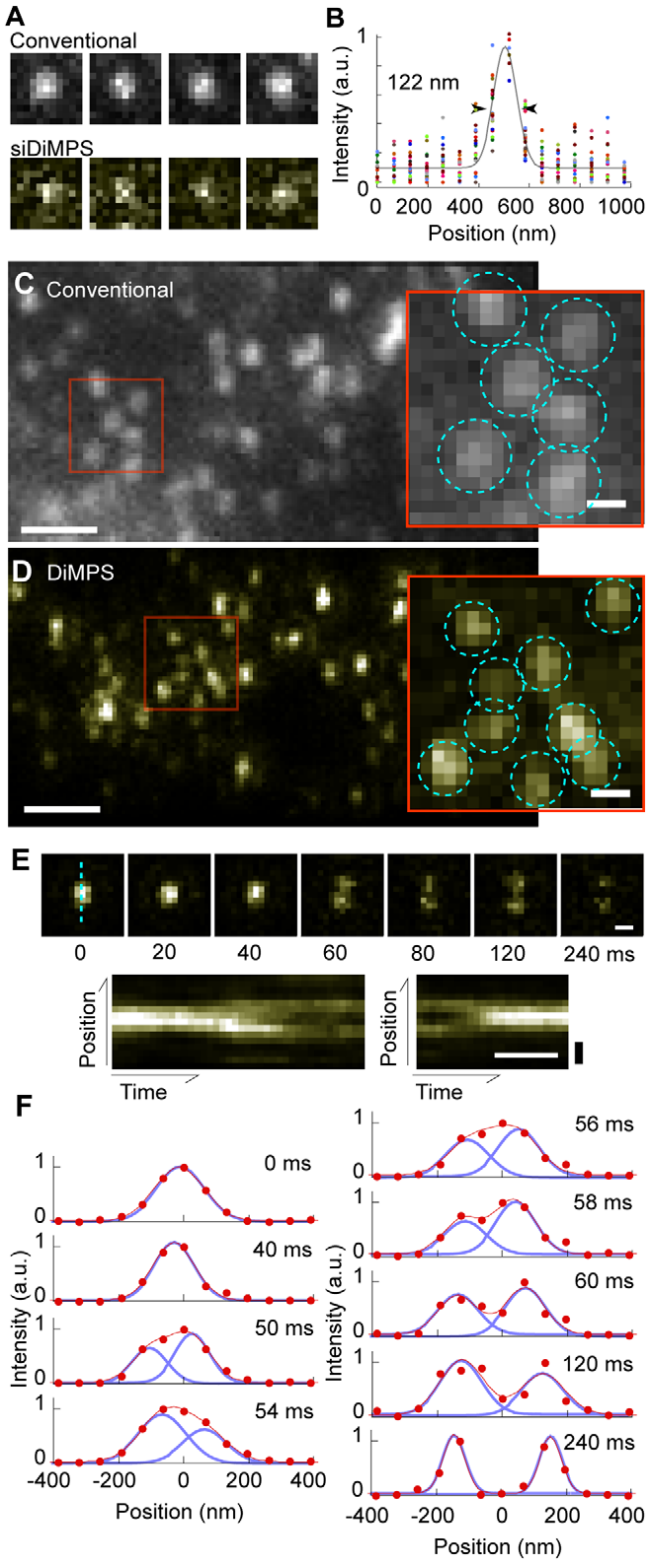
Individual quantum dots observation with 2 ms temporal resolutions with the siDiMPS. (A) Four typical fluorescence images of QDs captured by a high speed CCD camera with a conventional microscope (upper) and the DiMPS (lower). One pixel, 64.5 nm. (B) Ten PSFs of fluorescence QD images acquired with the DiMPS with 2 ms temporal resolution. The gray line is the fitted Gaussian curve, which indicates the spatial resolution (122 nm). (C, D) Fluorescent images of E-cadherin-QD on the cell membrane acquired by conventional microscopy (C) and DiMPS (D). Scale bars, 1 µm. Inserts are magnifications of the red rectangles. Scale bars, 200 nm. Dotted cyan circles indicate individual QDs. (E) Time courses of the dissociation of the E-cadherin complex (upper) and kymograph of the cyan broken line showing dissociation (lower left) and association (lower right). Numbers in the panels indicate elapsed time (ms). Position scale bars, 200 nm; time scale bar, 100 ms. (F) Time course of the cross-sections (cyan broken line) for the upper panel in E. Red and blue lines are fitting results using double Gaussian functions and two single Gaussian functions used to make the fitted double Gaussian, respectively.

Frame rate of the imaging system with DiMPS is defined by the frame rate of the camera. However, increasing the camera frame rate results in reduction of the S/N ratio because of the lack of photons the camera receives. The subtraction process in siDiMPS further reduces the S/N ratio [21]. We tested the effect of siDiMPS by increasing the camera frame rate with a quantum dot (QD), which is a semi-conductor fluorescent particle exhibiting intense and stable fluorescence [34]. Though the S/N ratio was obviously decreased by the siDiMPS process, FWHM improved to 100∼140 nm (122±17 nm) from 205 nm with a high-speed CCD camera at a frame rate of 2 ms (1.93 ms exposure time) when the individual QDs were fixed on a glass surface (Fig. 5, *A* and *B*).

We then observed the diffusive movements of individual E-cadherin labeled with QD on a cell membrane at 120 nm spatial and 2 ms temporal resolutions (Fig. 5, *C, D*, movie S8 and S9). More QDs could be independently identified in DiMPS than in conventional microscopy (Fig. 5, *C, D*, *inserts* and Fig. S4). Additionally, we could detect the association-dissociation cycle of an E-cadherin complex (Fig. 5, *E* and movie S10). By examining the time course of the distance between two QDs, which was obtained by fitting two Gaussian functions to one-dimensional cross sections, we estimated the dissociation velocity to be 8.7 nm/ms (Fig. 5, *F*). It is difficult to detect this rapid and small association-dissociation cycle with current superresolution methods because their temporal resolution is too low [11]. This observation shows the capability of siDiMPS, which allows 120 nm spatial resolution at 2 ms temporal resolution.

### On Site Fourier-filtering in Transmission Observation

**Figure 6:**
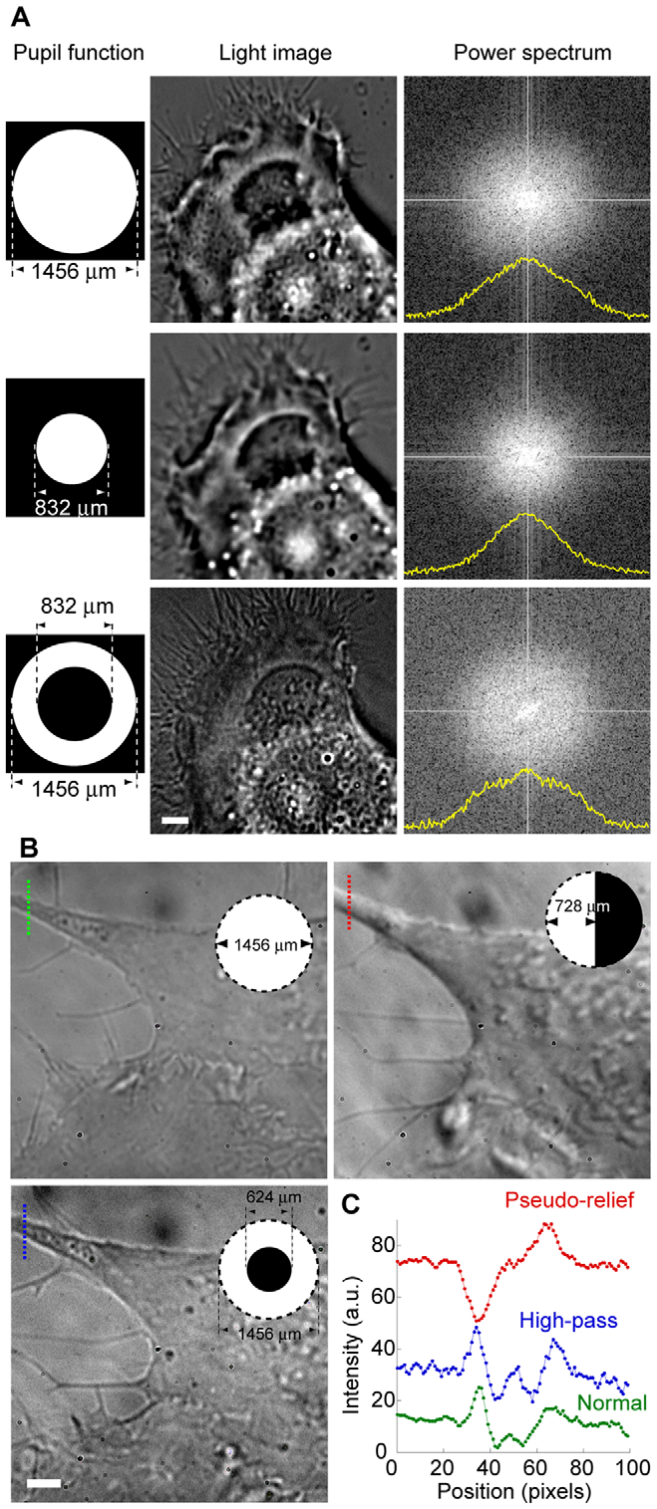
Transmission observation with DiMPS. (A) Effect of Fourier-filter with DiMPS. Left panels, the pupil functions used. White indicates transmission and black indicates blocking. Middle panels, images of a KPL4 cell on transmitted light irradiation. Right panels, two-dimensional power spectra of the middle images. Yellow lines are the intensity profile of the center of the 2D power spectrum, that is, the power spectrum along X direction. Scale bar, 2 µm. (B) Comparison of normal (left upper), pseudo-relief (right), and high-pass filtered (left lower) images. Inserts, the pupil functions used. (*C*) One dimensional cross-section of the broken lines in **B**. Green, normal. Blue, high-pass. Red, pseudo-relief. Scale bars, 2 µm.

With the DiMPS, the mask pattern of the pupil function can be changed on the computer during the image acquisition, leading to the realization of on site optical Fourier filtering with arbitrary pupil functions. We can also apply the DiMPS to transmission imaging. We obtained modulated images of a living cell and the power spectrum of Fourier-transformed images with various pupil function to investigate the effect of Fourier-filtration by pupil function modulation on bright field transmission microscopy (Fig. 6 *A* and Fig. S5). With decreasing the diameter of transmission area of the pupil function, the obtained image loses the high spatial frequency information. On the other hand, on masking of the center of the pupil function, the low spatial frequency information was lost, resulting in enhanced resolution, which can be seen in observation of thin filopodia.

The DiMPS also allows imaging of phase gradient specimens similarly to in the case of Hoffman modulation contrast (HMC), with which one can obtain pseudo-relief images of specimens [35]. On HMC microscopy, a diaphragm with an off-axis slit is placed on the front pupil plane of the illumination condenser lens, and a Hoffman modulator, which has three regions of different neutral densities (100%, 15% and 1% light transmission), is set on the Fourier plane of the objective. With this configuration, one can obtain images composed of light scattered and refracted by the phase difference and thus realize imaging of phase samples with high contrast. The DiMPS cannot modulate the incident light, but is capable of yielding different neutral densities of the pupil function on the detection pathway. When the numerical aperture (NA) of the objective is larger than that of the condenser lens, DiMPS gives an image effect similar to MHC by masking half of the Fourier plane (Fig. 6, *B*). Unlike a commercial HMC microscope, the DiMPS can arbitrarily change the degree of the phase gradient by changing the pupil function without changing the modulator-slit plate (Fig. S6). While the high-pass filter with the DiMPS enhanced the thin filopodia and the edge of a cell and improved the contrast, pseudo-relief imaging enabled us to observe the cell as a shadowed image; especially the small vesicles could be clearly observed (Fig. 6, *B* and *C*, and movie S11,S12,S13). Since the DiMPS can modulate two distinct images simultaneously, the microscope user can simultaneously observe the two effects of the pupil function modulations on an image by projecting these images side by side onto a camera. Thus, the DiMPS can be applicable to the imaging of phase samples, such as cells, by transmission imaging without need of modifying the incident light.

## Discussion

To obtain a more applicable and user-friendly microscope, we developed an optical system that can modulate a suitable pupil function for the microscope user’s observation. Though the basic principle of pupil function modulation was reported several decades ago and has been widely used so far, its advantages have been restricted because the mask pattern that the operator can prepare in advance is limited [12]–[14]. It has been problematic that various aberrations in the optics including the objective lens have made it difficult to precisely calculate the PSF shape and design a mask pattern. In particular, it is perhaps impossible for a biologist who is not familiar with microscopy to pre-calculate the PSF of the microscope in use. We solved this problem by introducing a LCOS mirror, with which the pupil function can be arbitrarily modulated and optimized on site during observation of real specimens. Of course, the effect of pupil function modulation can be achieved with different objectives (60X with NA of 1.49 in our test) (Fig. S7). The DiMPS can acquire two images at the same time for which the pupil functions are individually modulated within a camera frame (Fig. 1 and Fig. S3, *A*). Thus, the operator can change the pupil function of one pathway while simultaneously observing a normal image. Possibly, the operator can simultaneously obtain a high-pass filtered image and a relief contrast one. The DiMPS works well when two distinct effects are needed, for example, for embryo transplantation.

The most remarkable finding in this study is siDiMPS. Theoretically, the effect of the resolution enhancement by siDiMPS is similar to that of the subtraction of a raw image from a low-passed filtered one through digital image processing with a high-pass filter. We investigated the difference between siDiMPS and a digital high-pass filter (Fig. S8). High frequency information is extracted by subtracting a low-pass filtered image from a normal one (Fig. S8 *B* and *C*). Since digital image processing restricts the filtering in two dimensions, the high-pass filtering effect on the longitudinal axis is lower than that in siDiMPS. The digital high-pass filter completely removes the low spatial frequency, while the resolution of the high spatial frequency is clearly enhanced (Fig. S8, *E* and *F*), because the negative intensities in the PSF are attributed to the lack of low frequency information. The weight of the subtraction was set to decrease these negative values and the resultant resolution was only slightly improved (Fig. S8, *D* and *G*). The siDiMPS image retained the almost of low frequency information and the resolution was enhanced (Fig. S8 *H*). Thus, siDiMPS enhances the spatial resolution two-fold along both the longitudinal and transverse axes with slight loss of the lower frequency information.

There are a number of superresolution techniques [11], including stimulated emission depletion (STED) microscopy [36], saturated excitation (SAX) microscopy [37], structured illumination microscopy (SIM) [38], stochastic optical reconstruction microscopy (STORM) [39], fluorescence photoactivation localization microscopy (FPALM) [40], [41], and superresolution optical fluctuation imaging (SOFI) [42]. All of these methods are means of overcoming the diffraction limit and are becoming powerful tools for biological research. These methods require complicated equipment or long observation time to obtain superresolution images. In some circumstances, a simpler method that enhances high spatial frequency information is sufficient for more detailed observation even low spatial frequency information is lost. For example, siDiMPS works well for single particle tracking for a number of reasons [43], [44]. First, it can detect nano-particle or protein diffusions at speed too fast for the current superresolution methods. Second, shrinking the PSF increases the observable number of particles per unit area. Third, it requires less consideration of the side lobe. The siDiMPS does not physically overcome the diffraction limit of light, but achieved 120 nm spatial resolution within 2 ms exposure (Fig. 5 *A*). No current superresolution methods have achieved such a high temporal resolution. Also, the advantage of siDiMPS is compatibility with any fluorescent probe and the experimenter can observe the resolution-enhanced image in real-time and on site. The siDiMPS is not a superresolution but a pseudo-superresolution technique developed while focusing on temporal resolution.

**Figure 7:**
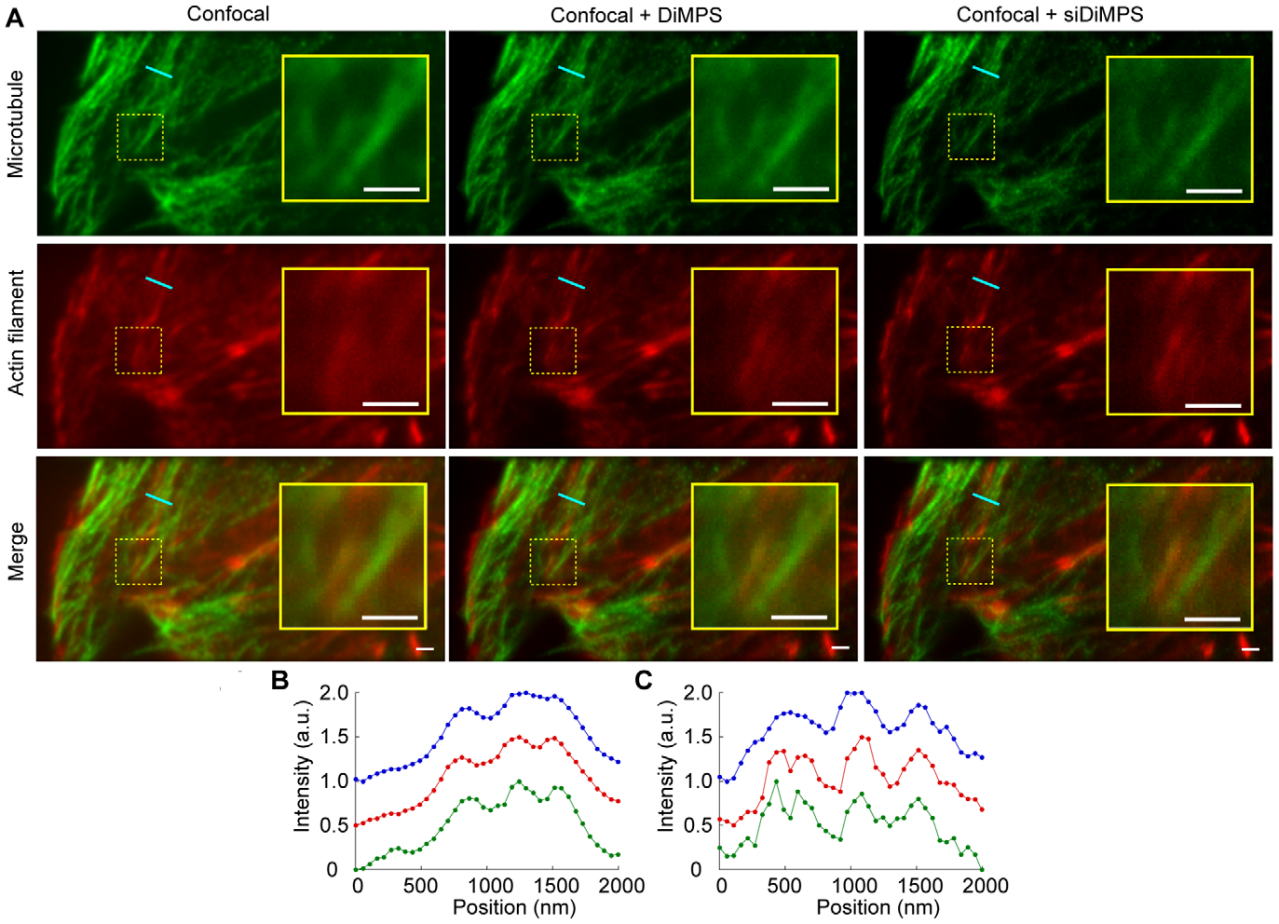
Confocal and multi-color imaging with the DiMPS. (A) Comparison of a confocal image (left), a high-pass filtered image obtained with the DiMPS combined with a confocal unit (middle), and siDiMPS combined with a confocal unit (right). Top, microtubules. Middle, actin bundles. Bottom, merged images of microtubules and actin bundles. Scale bars, 1 µm. Insets are enlarged images of the dotted yellow rectangles. (B, C) One dimensional fluorescence intensity profiles of the cyan lines in (A). (B) Microtubule. (C) Actin bundles. Blue, Red, and green lines were obtained from confocal, confocal + DiMPS, and confocal + siDiMPS images, respectively.

The DiMPS is based on relay optics. Therefore, the DiMPS can be combined with another microscopic instrument. To demonstrate the versatility of the DiMPS, we here show an example; combination of a spinning-disk confocal microscope and multi-color imaging (Fig. 7). The superresolving-mask of the DiMPS can shrink the PSF obtained with a spinning-disk confocal microscope, and siDiMPS gives stronger enhancement of the resolution (Fig. 7 *A*). The effect of the resolution enhancement was observed in both the green and red channels (Fig. 7 *B* and *C*). However, the fluorescent intensities were decreased by the spinning-disk confocal unit, and the siDiMPS further reduced the S/N ratio. To obtain a clearer image, the exposure time should be increased, which equals a decrease in the temporal resolution. We have to consider the best combination of S/N ratio and temporal resolution when using siDiMPS.

In conclusion, the DiMPS is thought to have the significant advantage that it is compatible with live cell imaging techniques because it is a relatively simple way to achieve various optical effects, including high-temporal resolution by the use of conventional equipment and/or fluorescent probes. The DiMPS can be constructed by using only relay optics, which allows the DiMPS to be set up beside a conventional fluorescence microscope. The temporal resolution and the size of the field of view are determined by the imaging device. Thus, the DiMPS shows great promise as a flexible optical microscopy technique in biological research fields.

## Materials and Methods

### Microscopy

Our microscope setup consisted of an epi-fluorescence microscope (IX-71; Olympus Co., Japan), an objective lens (150X PlanApo, 1.45 NA; Olympus Co., Japan), a relay optics box for dual-view imaging (GA03; G-Angstrom, Japan), and an electron multiplier type CCD camera (EM-CCD, iXon DV887 or DU897; Andor Technology plc., UK). In the relay optics box, the image from the microscope was relayed two times with two 4f-optical systems (Fig. 1). Reflective liquid crystal micro mirror arrays (LCM; DILA-SX073-S; JVC KENWOOD Corporation, Japan) have specifications of 1408×1058 resolution with a 10.4 µm pixel pitch. The contrast ratio of the LCM is 1000∶1. The LCMs were controlled by an Analog/Digital transfer board (D-ILA Evaluation Board, DEB-D4; Victor, Japan). The focal length of the lenses in the DiMPS were 75 mm for L1, 100 mm for L2, 70 mm for L3 and L4, and 130 mm for L5 (see Fig. 1). The total magnification of our setup was ∼370. A quartz depolarizer (DEQ-2S; Sigma Koki, Japan) was set at the back of the objective to negate any effect caused by the anisotropic polarization property of fluorescent probes. The position of the objective can be precisely controlled by a piezo actuator (P-721.CDQ and E-665.CR; Physik Instrumente, Germany). For confocal and multi-color imaging, a scanning confocal unit (CSU-X1; Yokogawa Electric Corporation, Japan) was placed at the image plane of the microscope. The DiMPS was set on the image plane of the scanning confocal unit.

The optical components were purchased from Sigma Koki Co., LTD (Japan), and the holders and stages from Suruga Seiki Co., LTD (Japan). Images obtained with the CCD camera were composed of 14-bit data per pixel. The images were imported into analysis software programmed in Visual C++ (Microsoft, USA) and the OpenCV library (IBM, USA).

### Biological Sample and Observation Conditions

For fluorescence imaging, an ∼30×30 µm^2^ area was illuminated with a xenon lamp with an excitation filter of 460∼495 nm. An actin bundle of a mouse fibroblast (L cell, gift from Prof. M. Takeichi) [45] fixed on a glass bottom dish was labeled with Alexa-488 phalloidin. For real time imaging, we used a breast cancer cell, a KPL cell, kindly provided from Prof. J. Kurebayashi [46], that overexpresses EB1 fused with GFP. The exposure time for the camera (iXon DV887) was 200 ms for actin observation and 1.0 s for EB1 observation.

For confocal and multi-color imaging, actin bundles and microtubules were labeled with rhodamine-phalloidin and Alexa488-anti-tubulin in a HeLa cell (purchased from Riken Cell Bank; RCB0007). An ∼30×30 µm^2^ area was simultaneously illuminated with blue (488 nm) and green (532 nm) lasers. The exposure time for the camera (iXon DV887) was 300 ms.

We used two types of cells for transmission imaging. One was a KPL4 cell, as a normal thick specimen. The other is myoblast cell line C2C12 (purchased from Riken Cell Bank; RCB0987), which is a useful model cell line for studying the differentiation of non-muscle cells into skeletal muscle cells [47], which was used as a thin specimen in our study. The light irradiation was performed with light of 460∼480 nm wavelength though a band-pass filter in a halogen lamp. The exposure time for the camera (iXon DV887) was 100 ms.

### siDiMPS Observation

We used an E-cadherin system for juxtamembrane observation. To fuse E-cadherin with GFP, we inserted mouse E-cadherin cDNA into a pCA-sal-EGFP-IRES-neo vector in which a neomycin resistance gene was driven by the internal ribosome entry sites (IRES) [48]. pCA-E-cadherin-EGFP-IRES-neo plasmids were transfected into mouse fibroblasts (L cells) by using an Amaxa electroporation system (Amaxa) and selected with 400 µg/ml G418 (EEG-L cells, gift from Prof. M. Takeichi) [45]. Just before observation of E-cadherin-EGFP localization, EEG-L cells were re-plated onto E-cadherin coated glass and cultured for 8–10 hours at 37°C in L-15 medium (Invitrogen). The E-cadherin coated glass was obtained as follows; goat anti-Human IgG-Fc (BETHYL) was nonspecifically bound to the surface of a glass bottom dish (Matsunami), followed by the loading of mouse E-cadherin-Fc (R&D Systems) into the dish. After incubation for 1 hour at 37°C, the glass surface was washed out with PBS and then blocked with 1% BSA containing 5 mM Ca^2+^ for 1 hour at 37°C. Microscopic observations were carried out at 25°C. The exposure time for the camera (iXon DV887) was 30.28 ms. To construct a final image, 100 images were averaged (total frame rate, 0.3 Hz).

For intracellular observation, we choose an autophagy system. The plasmid used for microtubule associated protein light chain 3 (LC3)-fused GFP (GFP-LC3) was kindly provided by Prof. T. Yoshimori (Department of Genetics Graduate School of Medicine, Osaka University) and Dr. S. Yamaoka (Tokyo Medical and Dental University). Mouse embryonic fibroblasts expressing GFP-LC3 were prepared as described previously [49]. Cells were cultured in Dulbecco’s modified Eagle’s medium (DMEM) supplemented with 10% fetal calf serum. For starvation, DMEM was replaced with phenol red-free Hank’s balanced salt solution, with incubation for 30 min at 37°C. Microscopic observations were carried out at 25°C. The exposure time for the camera (iXon DV887) was 30.28 ms.

### Single Particle Tracking of E-cadherin

Before single particle tracking of E-cadherin, cell surface E-cadherins were labeled with anti-E-cadherin mAb for 1 hour at 37°C and then QD goat F(ab’) 2 anti-rat IgG conjugate (Invitrogen) for another hour at 37°C. The final concentration of QD was 200 pM in 2 ml medium. The peak wavelength of the QD fluorescent spectrum was 605 nm. Microscope observations were carried out at 25°C. Exposure time of the camera (iXon DV897) was 1.942 ms (frame rate, 2 ms).

The two dimensional position of an individual QD could be calculated by fitting its fluorescent image with a two dimensional Gaussian function as follows,
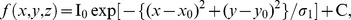
where I_0_ is the peak intensity, (x_0_, y_0_) is the position of the QD and C is the background intensity. When I_0_ was calculated with a fitting three times larger than the standard deviation of the background intensity, the computer could record the position (x_0_, y_0_) of the fluorescent spot.

## Supporting Information

Figure S1 **Modulation of the pupil function.** The pupil function is the reflection pattern in the liquid crystal mirror array. Black part shows where the light is blocked. The point spread function (PSF) is the fluorescence distribution of a φ100 nm bead with a peak emission spectrum at 515 nm (Invitrogen). Red lines show the one-dimensional fluorescence intensity profile of the PSF along the horizontal and optical axes. Gray lines are the one-dimensional fluorescence intensity profile of a normal PSF. Scale bars, 1 µm.
(TIF)

Figure S2 **Relationship between the mask-pattern and the intensity or the FWHM (full length half maximum).** (Left) The intensity (led) and FWHM of PSF in the transverse axis (blue) with various diameters using the apodizing-mask shown in Fig. S1, *left-top*. (Right) The intensity (led) and FWHM of PSF in the transverse axis (blue) with various inner diameters of the donut using the apodizing-mask shown in Fig. S1, *right-top*. The outer diameter is 1456 µm.
(TIF)

Figure S3 **Raw and displayed images with the DiMPS.** (A) Raw image of Alexa488-phalloidin-stained actin bundles in a fixed cell. Top, superresolving-masked image; bottom, apodizing-masked image. The images have 512×512 pixels (22×22 µm^2^). (B) Displayed image obtained by subtraction of the upper and lower images in A. The images were obtained with a 100 ms exposure time. Scale bar, 2 µm.
(TIF)

Figure S4 **Detection improvements of single particles by siDiMPS.** (**A**) Computer simulation of auto-detection of individual single fluorescent spots. This simulation used PSFs shown in Fig. 5A (upper; conventional microscope, lower, DiMPS). The fluorescent spots were placed randomly on 5×5 µm^2^ area whose pixel size was 64.5 nm. Value in each panel is the number of spots individually identified by our software shown in yellow circle (see method). (**B**) Relationship between the number of spots placed in the simulation and that of individually detected spots. Blue, conventional microscope. Red, DiMPS. Plots are the average and error bars are the standard deviation in 50 simulations. (**C**) Relationship between the number of spots set in the simulation and the percentage of individual detection. Blue, conventional microscope. Red, DiMPS. N = 50.
(TIF)

Figure S5 **Effect of Fourier-filter in transmission observation with DiMPS.** Left panels, the pupil functions used. White indicates transmit and black indicates blocking. Middle panels, images of a KPL4 cell on transmitted light irradiation. Right panels, power spectra of the middle images. Yellow lines are the intensity profile of the center of the power spectrum. Scale bar, 2 µm.
(TIF)

Figure S6 **Creating an MHC-like image with the DiMPS.** Pseudo-relief images obtained by masking half of the pupil function. Inserts, the pupil functions used.
(TIF)

Figure S7 **The DiMPS with a 60× objective.** (A) Modulation of the pupil function. Different reflection patterns in the liquid crystal mirror array were acquired with a 60× objective. The details are the same as in Fig. 2. (B) Point spread functions (PSFs) of the DiMPS (lower), and optimized DiMPS (upper)-acquired signals. Left panels, PSF images in the longitudinal-transverse plane. Middle and right panels, one-dimensional fluorescence intensity profiles of each PSF along the longitudinal axis (middle) and transverse axis (right). Gray lines indicate one-dimensional fluorescence intensity profiles of the non-masked PSF. (C) Immuno-fluorescence images of actin bundles obtained by conventional microscopy (top) and with the DiMPS (bottom). Insert, intensity profiles of the one-dimensional cross-sections (cyan lines) in each panel.
(TIF)

Figure S8 **Digital resolution enhancement (high-pass filter).** (A) Point spread functions (PSFs) after blurring with a Gaussian filter. First panel from the left, normal PSF (Normal); second, a PSF blurred with a Gaussian filter with a sigma of 2 pixels (SD-2); third, half the intensity of SD-2 (0.5×SD-2); and fourth, PSF blurred with a Gaussian filter with a sigma of 5 pixels (SD-5). Scale bars, 1 µm. (B-D) PSFs obtained by subtracting SD-2 image from Normal image (B); SD-5 image from Normal image (C); and 0.5×SD-2 from Normal image (D). Left panels, PSF images in the longitudinal-transverse plane. Middle and right panels, one-dimensional fluorescence intensity profiles of each PSF along the transverse axis (middle) and longitudinal axis (right), respectively (Red). Values indicate spatial resolution determined at full width at half maximum. Gray, one-dimensional cross-sections of Normal images. Scale bars, 1 µm. (E–H) Images acquired by conventional microscopy (E); by subtracting SD-2 from Normal image (F); by subtracting SD-5 from Normal image (G); and with the siDiMPS (H). Left panels, images of Alexa488-phalloidin stained actin bundles in a fixed cell. These images are the same as those in Fig. 3 G. Scale bars, 2 µm. Middle panels, enlarged images of the dotted yellow rectangles in the left panels. Scale bars, 0.5 µm. Right panels, one-dimensional fluorescence intensity profiles of the single yellow broken lines in the middle panels. Gray lines and circles, one-dimensional fluorescence intensity profiles of Normal images.
(TIF)

Movie S1 **Movie of EB1-GFP in a KPL4 cell obtained with a conventional fluorescent microscope.** The exposure time of the camera was 998 ms and the frame rate was 1.0 Hz. Pixel size, 43.2 nm. Movie speed was 15 times real time.
(AVI)

Movie S2 **Movie of EB1-GFP in a KPL4 cell obtained by extended PSF imaging.** The exposure time of the camera was 998 ms and the frame rate was 1.0 Hz. Pixel size, 43.2 nm. Movie speed was 15 times real time.
(AVI)

Movie S3 **Comparison of conventional microscope and siDiMPS images of E-cadherin-EGFP overexpressed in mouse fibroblasts.** Yellow indicates the siDiMPS images. The exposure time of the camera was 30.28 ms. The images shown are the averages of 100 acquired images (total frame rate, 0.3 Hz). Pixel size, 43.2 nm.
(AVI)

Movie S4 **Comparison of conventional microscope and the siDiMPS images of autophagosomes labeled with GFP-LC3 in mouse embryonic fibroblasts.** Yellow indicates the siDiMPS images. The exposure time of the camera was 30.28 ms. The images shown are averages of over 10 acquired images (total frame rate, 3 Hz). Pixel size, 43.2 nm.
(AVI)

Movie S5 **Montage of the movement of a small organelle on time lapse imaging by siDiMPS.** The exposure time of the camera was 30.28 ms. The images shown are averages of over 10 acquired images. The time lapse is 10 s. Pixel size, 43.2 nm.
(AVI)

Movie S6 **Montage of the amoeba-like movement of an autophagosome on time lapse imaging by siDiMPS.** The exposure time of the camera was 30.28 ms. The images shown are averages of over 10 acquired images. The time lapse is 10 s. Pixel size, 43.2 nm.
(AVI)

Movie S7 **Real time imaging of autophagosomes with 300 ms temporal resolution by siDiMPS.** The exposure time of the camera was 298 ms and the frame rate was 3 Hz. Pixel size, 43.2 nm. The movie speed was ten times real time.
(AVI)

Movie S8 **Real time imaging of Qdot-E-cadherin with 2 ms temporal resolution by a conventional microscope.** The exposure time of the camera was 1.942 ms and the frame rate was 500 Hz. Pixel size, 65.2 nm. Movie speed is 1.5 times real time.
(AVI)

Movie S9 **Real time imaging of Qdot-E-cadherin with 2 ms temporal resolution by siDiMPS.** The exposure time of the camera was 1.942 ms and the frame rate was 500 Hz. Pixel size, 65.2 nm. Movie speed is 1.5 times real time.
(AVI)

Movie S10 **Dissociation and association of a Qdot-E-cadherin dimer as seen by siDiMPS.** The exposure time of the camera was 1.942 ms and the frame rate was 500 Hz. Pixel size, 65.2 nm. Movie speed is 1.5 times real time.
(AVI)

Movie S11 **Movie of a HeLa cell obtained with a conventional light microscope.** The exposure time of the camera was 100 ms and the frame rate was 0.2 Hz. Pixel size, 43.2 nm. The movie speed was 300 times real time.
(AVI)

Movie S12 **Movie of a HeLa cell obtained on high-pass filtered imaging.** The exposure time of the camera was 100 ms and the frame rate was 0.2 Hz. Pixel size, 43.2 nm. The movie speed was 300 times real time.
(AVI)

Movie S13 **Movie of a HeLa cell obtained on pseudo-relief imaging.** The exposure time of the camera was 100 ms and the frame rate was 0.2 Hz. Pixel size, 43.2 nm. The movie speed was 300 times real time.
(AVI)
