# Supplementary figures and images for: Distinct Modulated Pupil Function System for Real-Time Imaging of Living Cells
Source: PLoS One. 2012 Sep 4;7(9):e44028. doi: 10.1371/journal.pone.0044028 (PMC3433489; doi:10.1371/journal.pone.0044028)

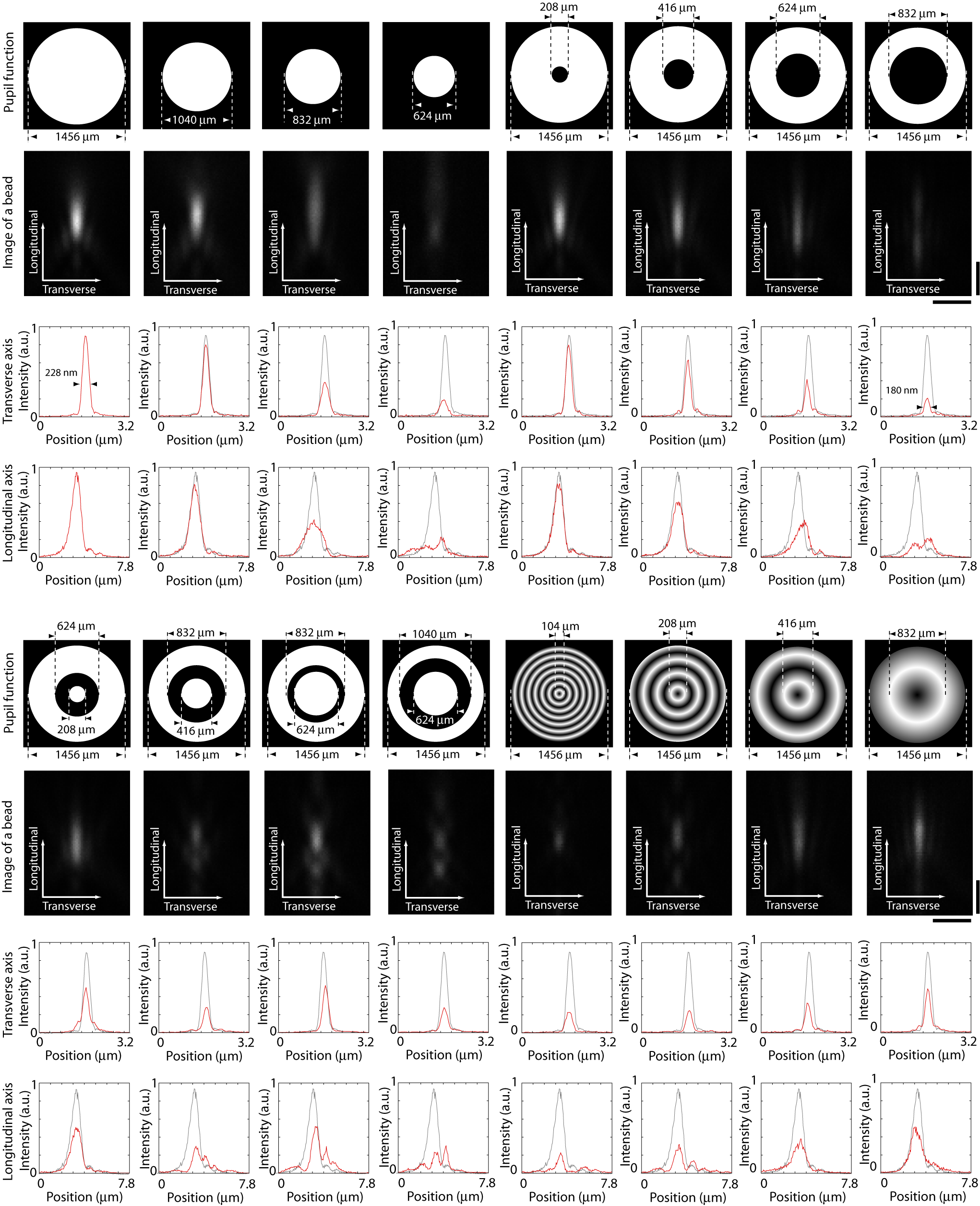

Supplement: Figure S1 — Modulation of the pupil function. The pupil function is the reflection pattern in the liquid crystal mirror array. Black part shows where the light is blocked. The point spread function (PSF) is the fluorescence distribution of a φ100 nm bead with a peak emission spectrum at 515 nm (Invitrogen). Red lines show the one-dimensional fluorescence intensity profile of the PSF along the horizontal and optical axes. Gray lines are the one-dimensional fluorescence intensity profile of a normal PSF. Scale bars, 1 µm. (TIF) [file pone.0044028.s001.tif]

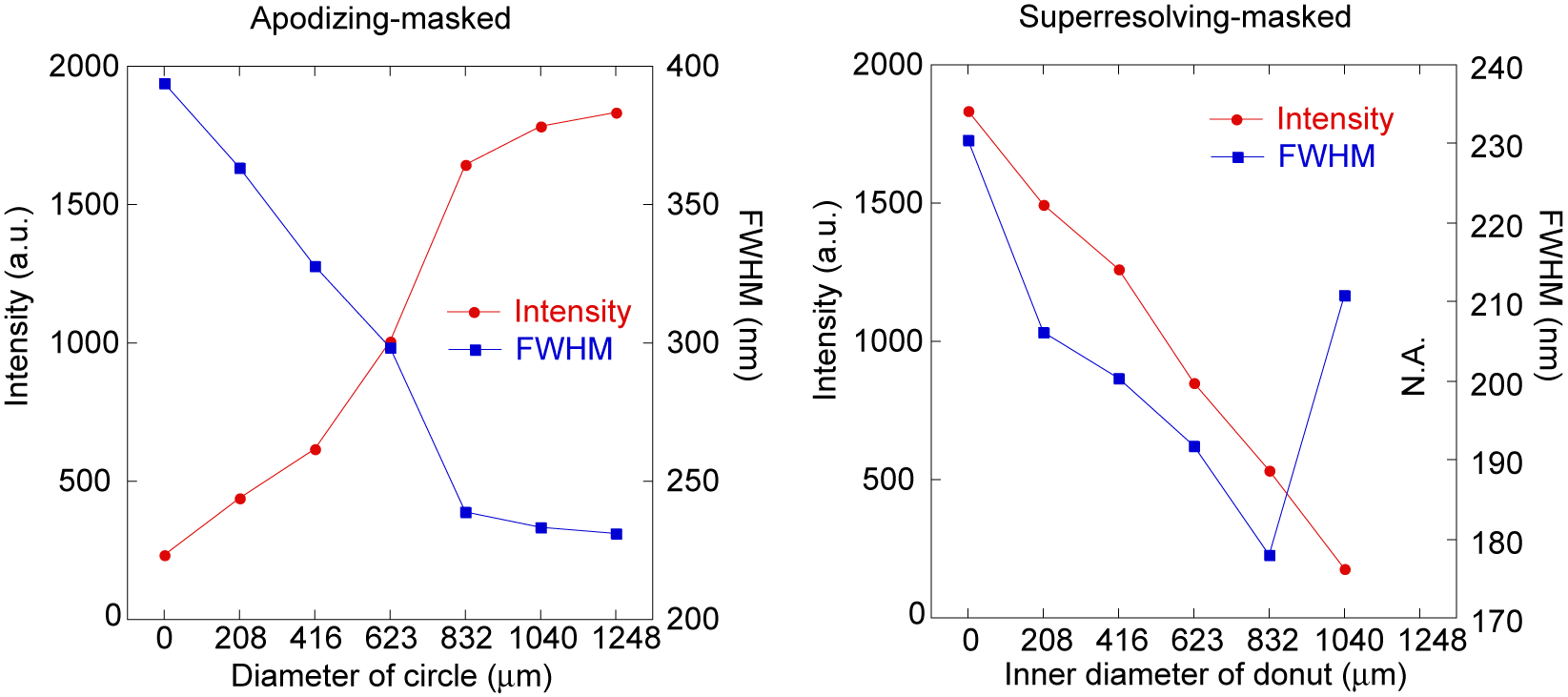

Supplement: Figure S2 — Relationship between the mask-pattern and the intensity or the FWHM (full length half maximum). (Left) The intensity (led) and FWHM of PSF in the transverse axis (blue) with various diameters using the apodizing-mask shown in Fig. S1, left-top. (Right) The intensity (led) and FWHM of PSF in the transverse axis (blue) with various inner diameters of the donut using the apodizing-mask shown in Fig. S1, right-top. The outer diameter is 1456 µm. (TIF) [file pone.0044028.s002.tif]

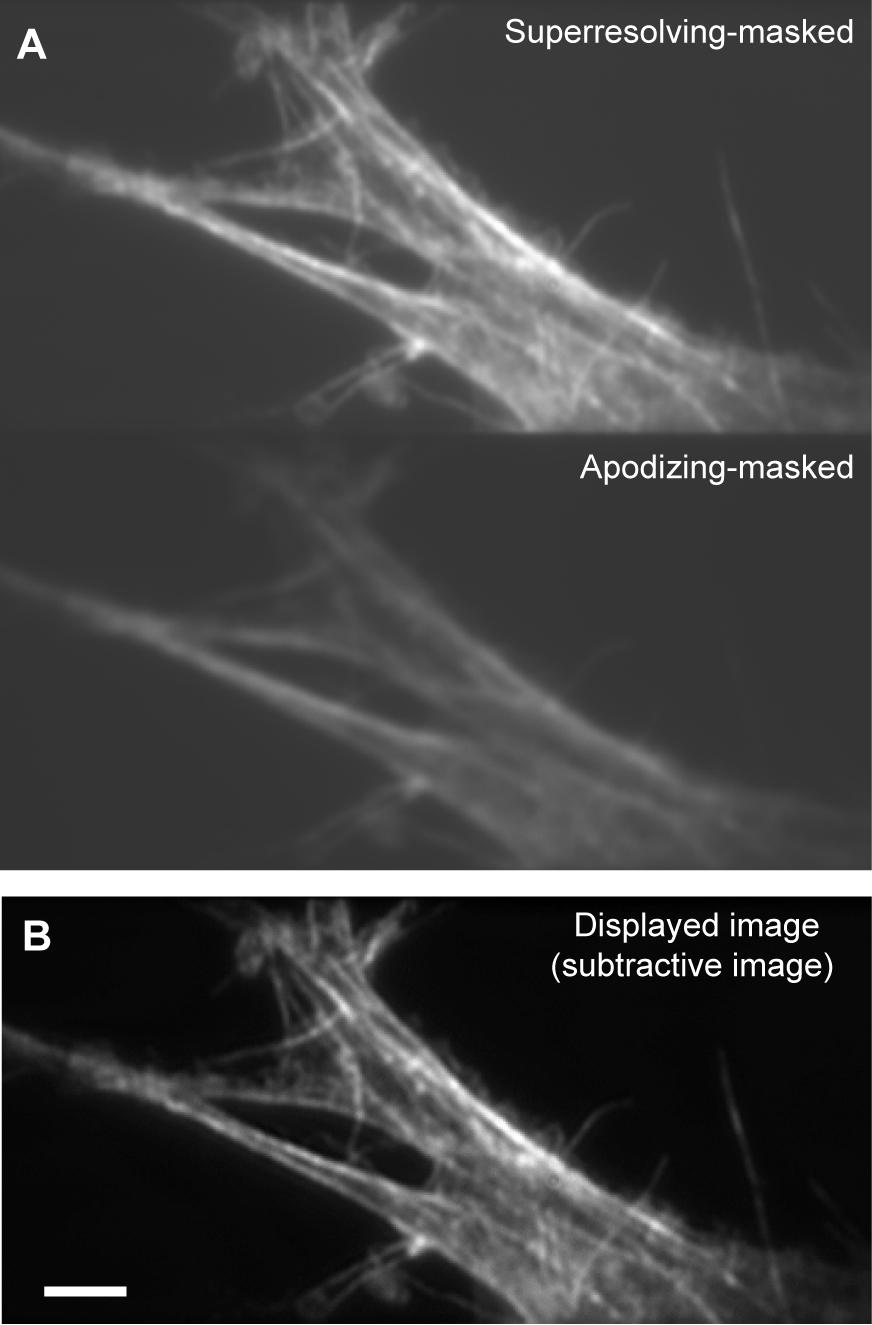

Supplement: Figure S3 — Raw and displayed images with the DiMPS. (A) Raw image of Alexa488-phalloidin-stained actin bundles in a fixed cell. Top, superresolving-masked image; bottom, apodizing-masked image. The images have 512×512 pixels (22×22 µm2). (B) Displayed image obtained by subtraction of the upper and lower images in A. The images were obtained with a 100 ms exposure time. Scale bar, 2 µm. (TIF) [file pone.0044028.s003.tif]

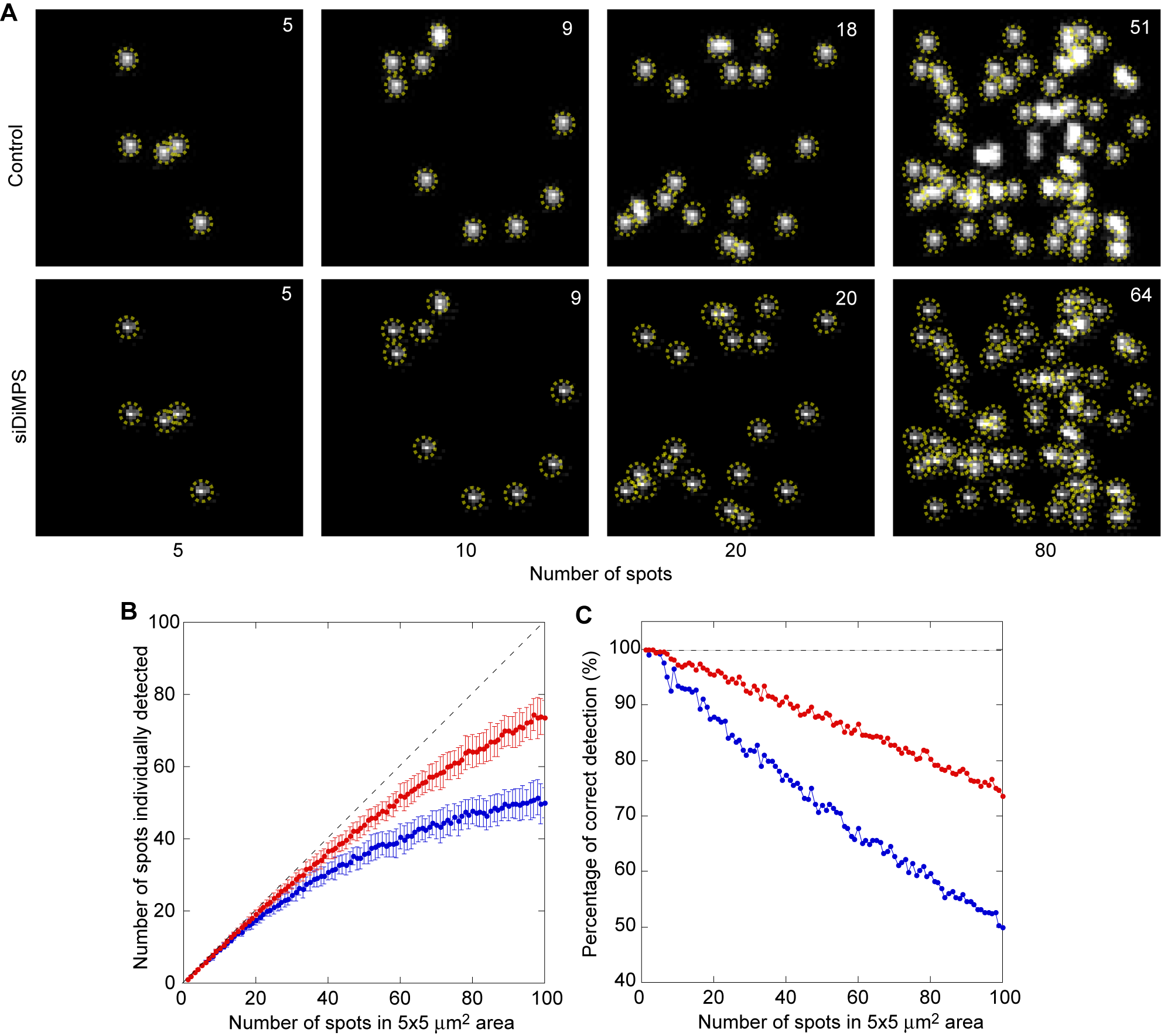

Supplement: Figure S4 — Detection improvements of single particles by siDiMPS. (A) Computer simulation of auto-detection of individual single fluorescent spots. This simulation used PSFs shown in Fig. 5A (upper; conventional microscope, lower, DiMPS). The fluorescent spots were placed randomly on 5×5 µm2 area whose pixel size was 64.5 nm. Value in each panel is the number of spots individually identified by our software shown in yellow circle (see method). (B) Relationship between the number of spots placed in the simulation and that of individually detected spots. Blue, conventional microscope. Red, DiMPS. Plots are the average and error bars are the standard deviation in 50 simulations. (C) Relationship between the number of spots set in the simulation and the percentage of individual detection. Blue, conventional microscope. Red, DiMPS. N = 50. (TIF) [file pone.0044028.s004.tif]

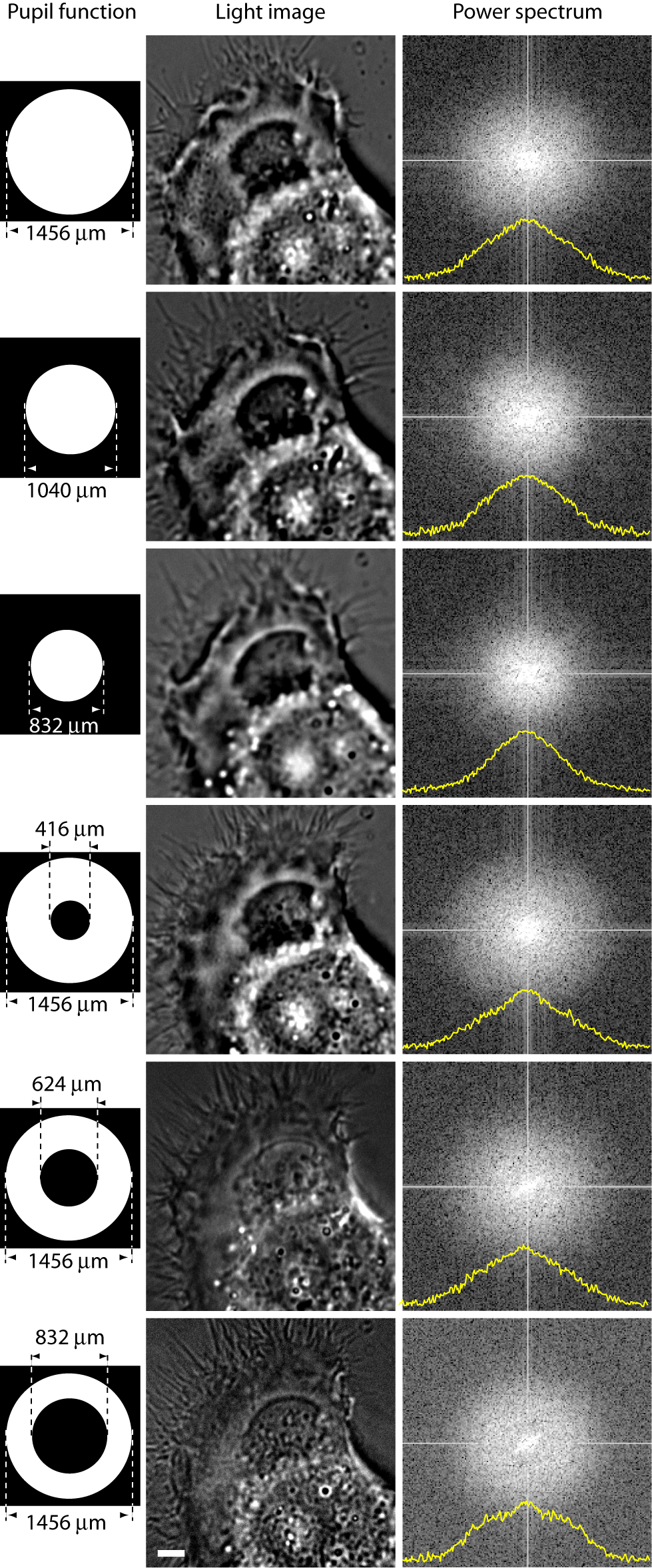

Supplement: Figure S5 — Effect of Fourier-filter in transmission observation with DiMPS. Left panels, the pupil functions used. White indicates transmit and black indicates blocking. Middle panels, images of a KPL4 cell on transmitted light irradiation. Right panels, power spectra of the middle images. Yellow lines are the intensity profile of the center of the power spectrum. Scale bar, 2 µm. (TIF) [file pone.0044028.s005.tif]

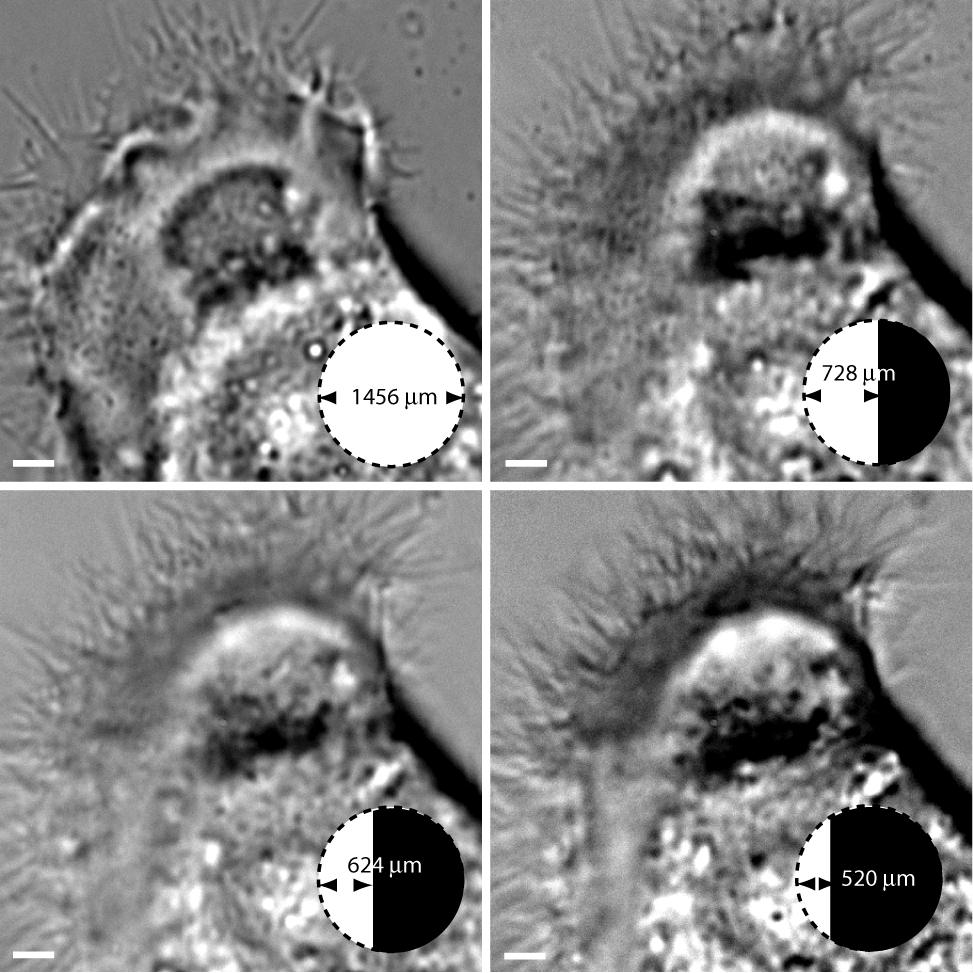

Supplement: Figure S6 — Creating an MHC-like image with the DiMPS. Pseudo-relief images obtained by masking half of the pupil function. Inserts, the pupil functions used. (TIF) [file pone.0044028.s006.tif]

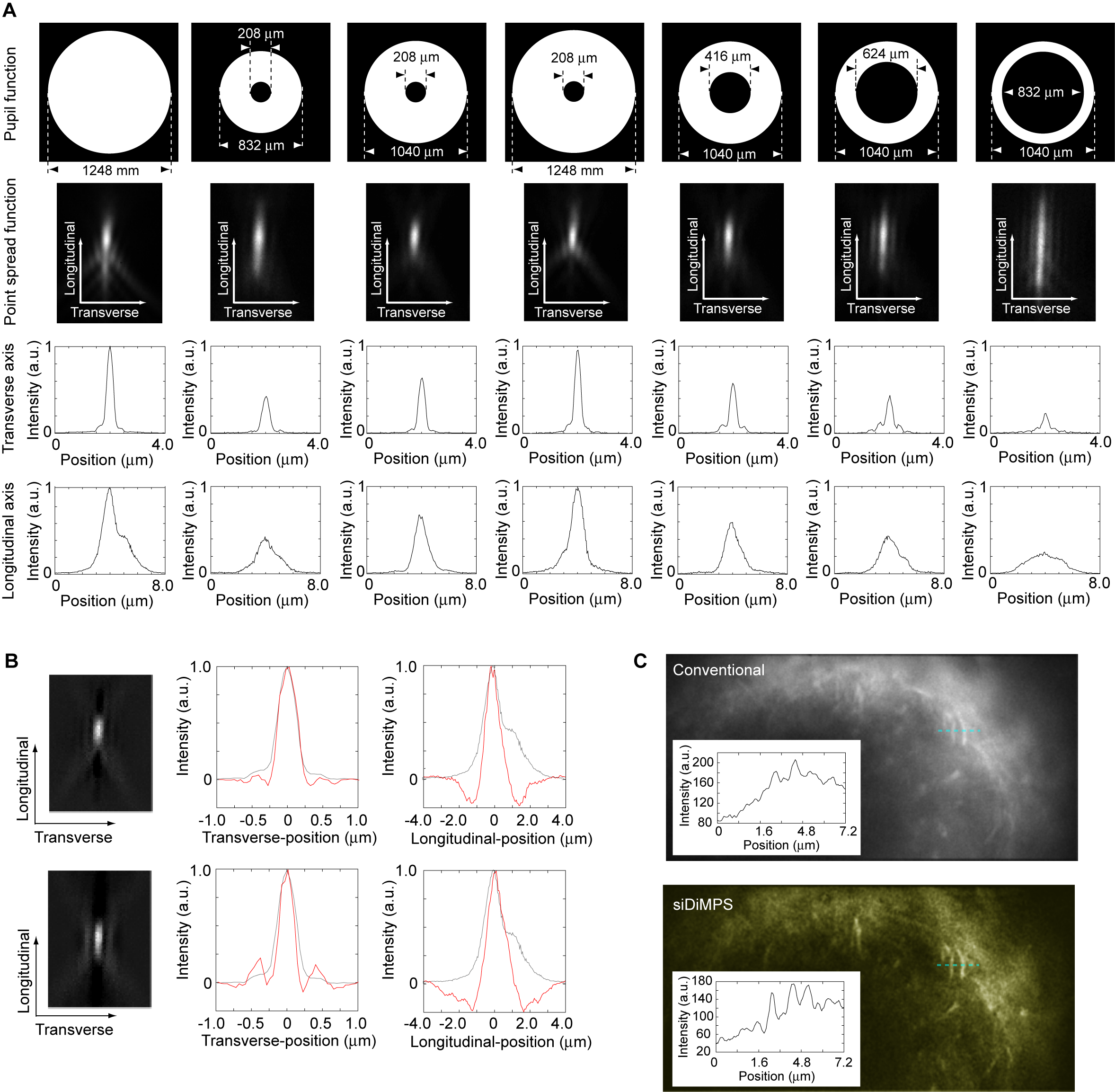

Supplement: Figure S7 — The DiMPS with a 60× objective. (A) Modulation of the pupil function. Different reflection patterns in the liquid crystal mirror array were acquired with a 60× objective. The details are the same as in Fig. 2. (B) Point spread functions (PSFs) of the DiMPS (lower), and optimized DiMPS (upper)-acquired signals. Left panels, PSF images in the longitudinal-transverse plane. Middle and right panels, one-dimensional fluorescence intensity profiles of each PSF along the longitudinal axis (middle) and transverse axis (right). Gray lines indicate one-dimensional fluorescence intensity profiles of the non-masked PSF. (C) Immuno-fluorescence images of actin bundles obtained by conventional microscopy (top) and with the DiMPS (bottom). Insert, intensity profiles of the one-dimensional cross-sections (cyan lines) in each panel. (TIF) [file pone.0044028.s007.tif]

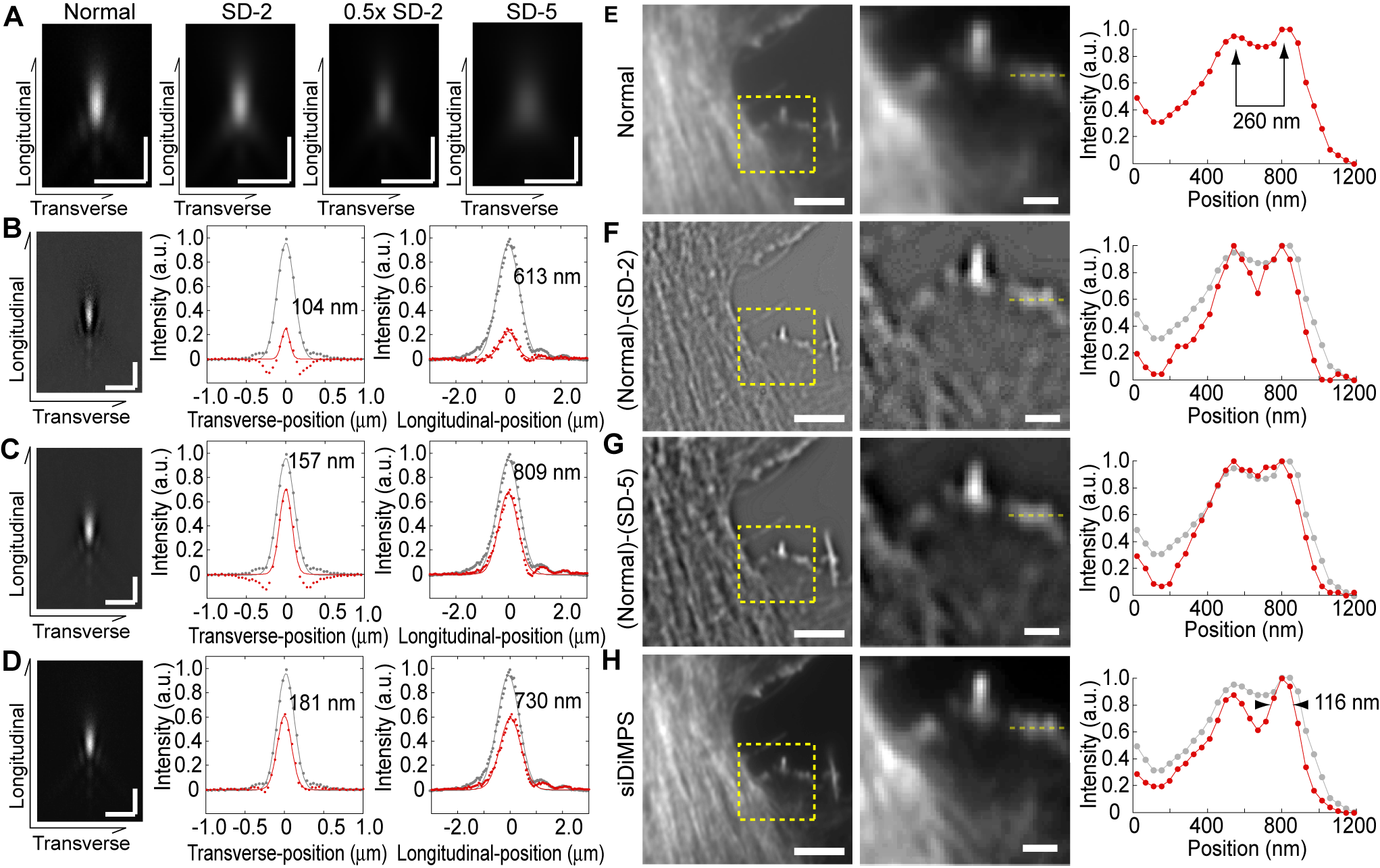

Supplement: Figure S8 — Digital resolution enhancement (high-pass filter). (A) Point spread functions (PSFs) after blurring with a Gaussian filter. First panel from the left, normal PSF (Normal); second, a PSF blurred with a Gaussian filter with a sigma of 2 pixels (SD-2); third, half the intensity of SD-2 (0.5×SD-2); and fourth, PSF blurred with a Gaussian filter with a sigma of 5 pixels (SD-5). Scale bars, 1 µm. (B-D) PSFs obtained by subtracting SD-2 image from Normal image (B); SD-5 image from Normal image (C); and 0.5×SD-2 from Normal image (D). Left panels, PSF images in the longitudinal-transverse plane. Middle and right panels, one-dimensional fluorescence intensity profiles of each PSF along the transverse axis (middle) and longitudinal axis (right), respectively (Red). Values indicate spatial resolution determined at full width at half maximum. Gray, one-dimensional cross-sections of Normal images. Scale bars, 1 µm. (E–H) Images acquired by conventional microscopy (E); by subtracting SD-2 from Normal image (F); by subtracting SD-5 from Normal image (G); and with the siDiMPS (H). Left panels, images of Alexa488-phalloidin stained actin bundles in a fixed cell. These images are the same as those in Fig. 3 G. Scale bars, 2 µm. Middle panels, enlarged images of the dotted yellow rectangles in the left panels. Scale bars, 0.5 µm. Right panels, one-dimensional fluorescence intensity profiles of the single yellow broken lines in the middle panels. Gray lines and circles, one-dimensional fluorescence intensity profiles of Normal images. (TIF) [file pone.0044028.s008.tif]
